# Disrupted Dynamic Functional Connectivity in Distinguishing Subjective Cognitive Decline and Amnestic Mild Cognitive Impairment Based on the Triple-Network Model

**DOI:** 10.3389/fnagi.2021.711009

**Published:** 2021-09-17

**Authors:** Chen Xue, Wenzhang Qi, Qianqian Yuan, Guanjie Hu, Honglin Ge, Jiang Rao, Chaoyong Xiao, Jiu Chen

**Affiliations:** ^1^Department of Radiology, The Affiliated Brain Hospital of Nanjing Medical University, Nanjing, China; ^2^Institute of Brain Functional Imaging, Nanjing Medical University, Nanjing, China; ^3^Department of Rehabilitation, The Affiliated Brain Hospital of Nanjing Medical University, Nanjing, China; ^4^Institute of Neuropsychiatry, The Affiliated Brain Hospital of Nanjing Medical University, Nanjing, China

**Keywords:** subjective cognitive decline, amnestic mild cognitive impairment, dynamic functional connectivity, resting state functional magnetic resonance imaging, triple network

## Abstract

**Background:** Subjective cognitive decline and amnestic mild cognitive impairment (aMCI) were widely thought to be preclinical AD spectrum disorders, characterized by aberrant functional connectivity (FC) within the triple networks of the default mode network (DMN), the salience network (SN), and the executive control network (ECN). Dynamic FC (DFC) analysis can capture temporal fluctuations in brain FC during the scan, which static FC analysis cannot. The purpose of the current study was to explore the changes in dynamic FC within the triple networks of the preclinical AD spectrum and further reveal their potential diagnostic value in diagnosing preclinical AD spectrum disorders.

**Methods:** We collected resting-state functional magnetic resonance imaging data from 44 patients with subjective cognitive decline (SCD), 49 with aMCI, and 58 healthy controls (HCs). DFC analysis based on the sliding time-window correlation method was used to analyze DFC variability within the triple networks in the three groups. Then, correlation analysis was conducted to reveal the relationship between altered DFC variability within the triple networks and a decline in cognitive function. Furthermore, logistic regression analysis was used to assess the diagnostic accuracy of altered DFC variability within the triple networks in patients with SCD and aMCI.

**Results:** Compared with the HC group, the groups with SCD and aMCI both showed altered DFC variability within the triple networks. DFC variability in the right middle temporal gyrus and left inferior frontal gyrus (IFG) within the ECN were significantly different between patients with SCD and aMCI. Moreover, the altered DFC variability in the left IFG within the ECN was obviously associated with a decline in episodic memory and executive function. The logistic regression analysis showed that multivariable analysis had high sensitivity and specificity for diagnosing SCD and aMCI.

**Conclusions:** Subjective cognitive decline and aMCI showed varying degrees of change in DFC variability within the triple networks and altered DFC variability within the ECN involved episodic memory and executive function. More importantly, altered DFC variability and the triple-network model proved to be important biomarkers for diagnosing and identifying patients with preclinical AD spectrum disorders.

## Introduction

Alzheimer's disease is a great medical challenge that haunts the world because of its progressive, irreversible, and incurable nature (Jessen et al., 2014a,b). Subjective cognitive decline (SCD) is regarded to be the preclinical stage of AD, and amnestic mild cognitive impairment (aMCI) is considered to be the prodromal stage of AD, both of which have received more attention in recent years (Morris and Cummings, 2005; Jessen et al., 2014a,b; Xue et al., 2019). Patients with SCD, which refers to self-reported memory decline in elderly persons with normal objective cognitive performance, are widely believed to have two times to give rise to aMCI/Alzheimer's disease (AD) than elderly without SCD (Jessen et al., 2014b; Mitchell et al., 2014). Patients with aMCI, which is characterized by subjective memory decline, are thought to have nearly 10 times to progress to AD than healthy elderly people (Bischkopf et al., 2002; Jessen et al., 2014b; Chen et al., 2019; Slot et al., 2019). Due to the lack of effective treatment for AD, comparing and analyzing the neuroimaging characteristics of SCD and aMCI are crucial to research the early biomarkers of the preclinical AD spectrum.

Resting-state functional magnetic resonance imaging, which is a task-independent and powerful imaging modality, has been widely used to investigate the intrinsic functional connectivity networks of neuropsychiatric diseases (Li et al., 2002; Zhang and Raichle, 2010). Of the many intrinsic brain networks, the triple-network model, composed of the default-mode network (DMN), the salience network (SN), and the executive control network (ECN), has been the focus of recent research (Menon, 2011; Joo et al., 2016; Zhan et al., 2016). Numerous studies have suggested that the triple networks can be used to detect the reliability and stability of large-scale connections, which are damaged in neuropsychiatric diseases (He et al., 2014; Joo et al., 2016; Wu et al., 2016; Li et al., 2019). Moreover, the triple-network model provides a common framework for checking the reliable and stable patterns of large-scale connectivity (Menon, 2011). The DMN, mainly located in the ventromedial prefrontal cortex (vmPFC) and posterior cingulate cortex, is activated in internally directed cognitive activities, such as self-referential mental processes and social functions (Raichle et al., 2001; Broyd et al., 2009). The ECN, primarily involved in the lateral posterior parietal cortex and dorsolateral prefrontal cortex, is activated during externally directed higher-order cognitive function, including working memory, decision-making, and attention (Liang et al., 2016). The SN, which primarily includes the anterior cingulate cortex and anterior insula, is associated with affective processes, attention, and interoception (Sridharan et al., 2008; Uddin, 2015). Specifically, when salient events are detected, the SN can activate brain networks, direct the DMN and ECN to perform cognitive tasks, and help the corresponding brain regions to respond to stimuli appropriately (Menon and Uddin, 2010; Menon, 2011). Further study of triple-network model alterations in SCD and aMCI could help us better understand their pathological mechanisms.

Many neuroimaging studies have demonstrated that SCD and aMCI patients showed altered functional connectivity (FC) in the triple networks (Brier et al., 2012; Uddin, 2015; Chand et al., 2017). However, all the aforementioned studies were based on the assumption that the functional networks were spatiotemporally static during MRI scans (Chang and Glover, 2010). Due to the complexity and changing environment of the human brain, the assumption that brain activity remains static is too simplistic and may not reflect the dynamic characteristics of brain activation and connectivity (Preti et al., 2017). Previous rsfMRI studies have suggested that brain FC patterns can be time varying across a short time window; this phenomenon is known as dynamic FC (DFC) (Hutchison et al., 2013). DFC analysis has become an important tool in resting-state functional magnetic resonance imaging (rsfMRI) research by capturing temporal fluctuations in brain FC during the scan (Hutchison et al., 2013). Previous studies have demonstrated that the quantification of DFC disruption might be a sensitive biomarker or a prognostic indicator of disease progression and cognitive function (Long et al., 2019; Finc et al., 2020). Moreover, some studies have highlighted the potential role of DFC analysis in improving the accuracy of disease diagnosis, which made it necessary to apply DFC analysis to the diagnosis of AD spectrum disorders (Lei et al., 2020).

A number of studies found that AD showed altered DFC. Gu et al. suggested that AD showed decreased regional temporal variability, primarily in the temporal, parietal, and somatomotor regions (Gu et al., 2020). The authors also found that disrupted DFC was associated with cognitive function in patients with AD. They claimed that DFC analysis provided novel insight into the pathophysiological mechanisms of AD. In recent years, the research focus has been shifted to preclinical AD spectrum disorders, including SCD and MCI. Dong et al. found that patients with SCD showed both increased and decreased temporal variability compared with healthy controls (HCs) (Dong et al., 2020). Niu et al. found that patients with aMCI showed altered DFC in the prefrontal and parietal cortexes compared with HCs, and the regions were mainly in the DMN (Niu et al., 2019). Córdova-Palomera et al. suggested that patients with MCI showed altered DFC mainly in the frontal-superior, temporal, and default modes compared with patients with AD (Cordova-Palomera et al., 2017). However, the previous studies did not reveal changes in DFC with the progression of preclinical AD spectrum disorders. It is unclear whether there are common or specific changes in DFC features in SCD and aMCI. Specifically, there are few studies on alterations in DFC variability within the triple networks in patients with SCD and aMCI and their diagnostic value for SCD and aMCI.

Therefore, in the current study, using combined rsfMRI and the classic sliding time-window correlation approach, we aimed to reveal changes in DFC variability within the triple networks in patients with SCD and aMCI, as well as their relationship with cognitive function. We further explored the diagnostic efficiency of DFC variability in patients with SCD and aMCI. We hypothesized that DFC variability within the triple networks in patients with SCD and aMCI had varying degrees of change, and that altered DFC variability of the triple networks may contribute to cognitive decline. Additionally, a comprehensive analysis of DFC temporal variability within the triple networks might serve as an indicator to diagnose and identify SCD and aMCI.

## Materials and Methods

### Subjects

The applied research data were obtained from our in-home database: Nanjing Brain Hospital-Alzheimer's Disease Spectrum Neuroimaging Project (NBH-ADsnp) (Nanjing, China), which is continuously being updated. Related information of the NBH-ADsnp was summarized in *SI Methods*. The research gained approval by the responsible Human Participants Ethics Committee of the Affiliated Brain Hospital of Nanjing Medical University (No. 2018-KY010-01 and No. 2020-KY010-42). All volunteers participated voluntarily and with written informed consent. The current study used 151 data (until January 21, 2020), including 58 healthy control (HC), 44 SCD, and 49 aMCI from the NBH-ADnsp database. The inclusion and exclusion criteria of participants were provided in *SI Method*. All subjects underwent a comprehensive and standardized clinical evaluation interview, including demographic inventory, medical history, neurological and mental status examination, and MRI scan.

### Neurocognitive Assessments

Classical and comprehensive neurocognitive assessments were performed for all the participants, including general cognitive functions, episodic memory, executive function, information processing speed, and visuospatial function. Details of the neurocognitive assessments were summarized in *SI Methods*.

#### MRI Data Acquisition

The details of image acquisition parameters (structure MRI images and rsfMRI images) are provided in *SI Methods*.

### Preprocessing of rsfMRI Data

Functional MRI images were analyzed as described in previous studies using the DPABI based on the SPM program, implemented in MATLAB2013b with the following steps (Chen et al., 2016a, 2020): We discarded the first 10 volumes and performed slice-timing correction and head motion correction. The participants with excessive head motion (cumulative translation or rotation of > 3. mm or 3.^0^) were excluded. Subsequently, segmentation and nuisance covariate regression with 24 motion parameters, global signal, white matter signal, and cerebrospinal fluid signal were performed. Then, we selected a filtering frequency of 0.01–0.08 Hz, used segmented T1 image for normalization, and resampled to an isotropic voxel size of 3 mm. Finally, we applied spatial smoothing with a 6-mm full width at half-maximum Gaussian kernel and detrending.

**Figure 1:**
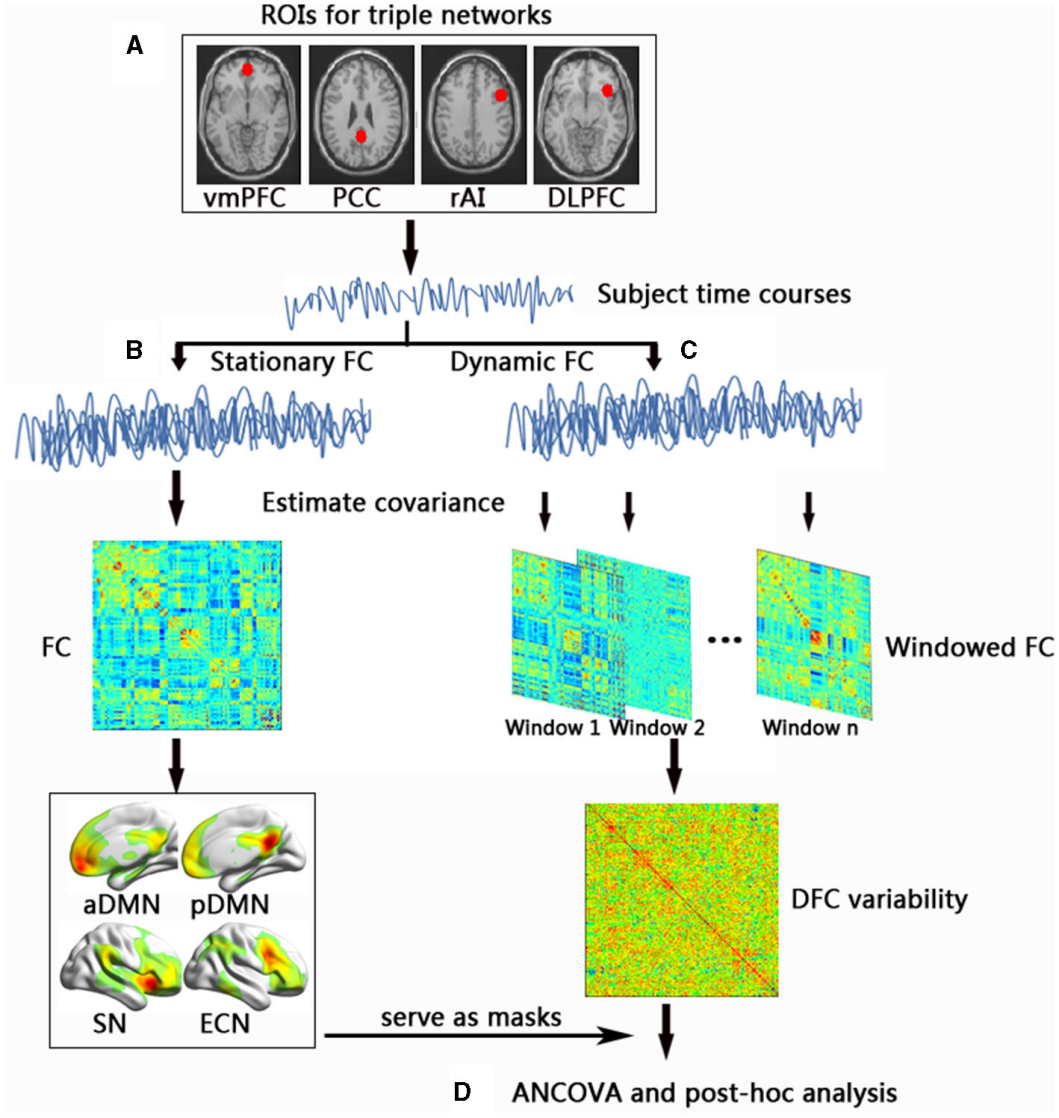
A flow chart for the dynamic functional connectivity analysis in this study. **(A)** For the RS-fMRI data of all subjects, we first used four ROIs to prepare for the next seed-based functional connectivity analysis. **(B)** Then, we adopted stationary functional connectivity analysis and obtained the template of the triple networks. **(C)** We applied the sliding window approach to analyze the dynamic functional connectivity of the obtained 96 windows. Afterward, we calculated dynamic functional connectivity variability across the windows. **(D)** Last, we performed the statistical analysis. ROI, regions of interests; vmPFC, ventromedial prefrontal cortex; PCC, posterior cingulated cortex; rAI, right anterior insula; DLPFC, dorsolateral prefrontal cortex; FC, functional connectivity; DFC, dynamic functional connectivity; aDMN, anterior default mode network; pDMN, posterior default mode network; SN, salience network; ECN, executive control network. ANCOVA, analysis of covariance.

After preprocessing, we further processed the preprocessed data according to the following steps illustrated in Figure 1.

### Definition of Functional Brain Networks

Seed-based static FC analysis was carried out to extract the triple networks. In the current study, four 10-mm spherical regions of interest (ROIs) centered in the vmPFC (MNI space: 0, 52, and −6) for the anterior DMN (aDMN), posterior cingulated cortex (PCC) (MNI space: 0, −53, and 26) for the posterior DMN (pDMN), right anterior insula (rAI) (MNI space: 38, 22, and −10) for the SN, and dorsolateral prefrontal cortex (MNI space: 48, 12, and 34) for the ECN were created according to previous studies (Wotruba et al., 2014; Chen et al., 2016b; Xue et al., 2019). The average time series of the ROIs in each participant was extracted, and voxel-wise cross-correlation analysis was conducted between the average time series within the ROIs and the whole brain within the GM mask. Fisher's z-transformation was applied to enhance the normality of the correlation coefficients.

Following this, the individual correlation maps from the HC group were subjected to random-effects analysis using a one-sample *t*-test. The threshold was set at a *p* < 0.05 with threshold-free cluster enhancement (TFCE) approach (1,000 random permutations) and family-wise error (FWE) correction. The regions with positive functional connections to the four ROIs were defined as templates for the aDMN, pDMN, SN, and ECN.

### Seed-Based DFC Variability Within the Triple Networks

The dynamic brain connectome analysis toolbox (http://restfmri.net/forum/DynamicBC) was used to compute DFC variability within the aDMN, pDMN, SN, and ECN. First, similar to the above static FC analysis, seed-based (vmPFC, PCC, rAI, and dorsolateral prefrontal cortex) voxel-wise DFC was applied to calculate DFC changes in the triple networks. The classic sliding time-window correlation method was used to compute the correlation between each ROI with a width of 40 TRs slid in steps of 2 TR according to previous studies, resulting in the analysis of 96 windows (Lin et al., 2018; Ma et al., 2019). Each obtained correlation coefficients were converted to a z score by the Fisher r-to-z transformation to improve normality. These Fisher's *z*-transformed correlation results were used to further calculate the temporal variation in DFC.

### Statistical Analysis

The Statistical Package for the Social Sciences (SPSS) software version 22.0 (IBM, Armonk, NY, USA) was used to analyze the demographic and clinical information. The analysis of covariance (ANCOVA) and chi-squared tests were conducted to compare the demographic and neurocognitive data across the three groups with SCD, aMCI, and HC. Bonferroni's correction with a *p* < 0.05 was used for *post hoc* analysis.

One-way ANCOVA was used to compare the differences in DFC variability in the aDMN, pDMN, SN, and ECN within the corresponding network mask among the three groups with SCD, aMCI, and HC after controlling for the effects of age, gender, and years of education. The non-parametric permutation test with the permutation times of 1,000 was performed in the present study to precisely control the false-positive rate. Corrected *p* < 0.05 and cluster numbers of ≥ 20 voxels (cluster size ≥ 540 mm^3^) were applied to multiple comparisons. The two-sample *t*-test was used for *post hoc* comparisons with the mask from the ANCOVA analysis and age, gender, and years of education as covariates. The significance level was set with a TFCE-FWE corrected *p* < 0.05 and a cluster number of > 9 voxels (cluster size > 243 mm^3^).

Significantly, altered DFC variability was extracted with the DPABI and used for the next correlation analysis. Correlation analysis was conducted by SPSS software to explore the relationship between altered DFC variability and cognitive domains with age, gender, and years of education as covariates (Bonferroni-corrected, *p* < 0.05).

### Binary Logistic Regression Analysis

Univariate and multivariable analyses of binary logistic regression were conducted in SPSS software to test the diagnostic value of DFC variability in SCD and aMCI. Altered DFC variability and cognitive function in univariate analysis were included in the multivariable models using backward elimination according to the likelihood ratio with a variable selection criterion of *p* < 0.05. We estimated the receiver-operating characteristic (ROC) curve and the area under the receiver-operating characteristic curve (AUC) to assess the predictive ability of the univariate and multivariable models according to the accuracy, sensitivity, and specificity. A *p* < 0.05 was considered statistically significant.

## Results

### Demographic and Neurocognitive Characteristics

The demographic and neurocognitive characteristics of all subjects, including 49 with aMCI, 44 with SCD, and 58 with HCs, are shown in Table 1. As is expected, the results showed significant differences in cognitive performance. The aMCI group showed significantly lower episodic memory (EM) and executive function (EF) scores compared with both the SCD and HC groups. The aMCI group showed significantly lower information processing speed and visuospatial function compared with the HC group (Bonferroni's *post hoc* correction, *p* < 0.05).

**Table 1:** Demographics and clinical measures of three groups, including SCD, aMCI, and HC.

	**HC(58)**	**SCD(44)**	**aMCI(49)**	***F*-values(χ^**2**^)**	***P*-values**
Age (years)	63.328 ± 6.28	66.000 ± 7.80	63.633 ± 7.58	1.966	0.144
Gender (male/female)	25/33	8/36^**^	13/36	7.865	0.020^c^
Education level (years)	12.40 ± 2.52	12.33 ± 2.59	10.99 ± 2.95^*^	4.359	0.014^a^
MMSE scores	28.62 ± 1.24	28.34 ± 1.14	27.22 ± 1.86^***^/^**^	13.319	<0.001^a^^b^
MDRS-2	141.57 ± 2.21	139.89 ± 3.61	136.80 ± 5.10^***^/^***^	11.808	<0.001^a^^b^
MoCA	25.11 ± 2.52	24.64 ± 1.95	22.77 ± 2.98^***^/^**^	21.740	<0.001^a^^b^
SCD-Q	3.51 ± 1.52	6.45 ± 0.89^***^	5.02 ± 1.92^***^/^***^	46.568	<0.001^c^^a^^b^
**Composite Z scores of each cognitive domain**					
Episodic memory	0.185 ± 0.080	0.330 ± 0.093	−0.519 ± 0.090^***^/^***^	25.178	<0.001^a^^b^
Information processing speed	0.190 ± 0.084	0.042 ± 0.098	−0.264 ± 0.095^**^	6.339	0.002^a^
Executive function	0.175 ± 0.065	0.147 ± 0.076	−0.330 ± 0.074^***^/^***^	15.166	<0.001^a^^b^
Visuospatial function	0.068 ± 0.098	0.226 ± 0.114	−0.218 ± 0.111^*^	3.954	0.021^b^

*Numbers are given as means ± standard deviation, SD unless stated otherwise. Values for age derived from ANCOVA; gender from chi-square test; all clinical measures from ANCOVA with age, gender, years of education as covariates. MMSE, mini-mental state examination; MDRS-2, Mattis Dementia Rating Scale-2; MoCA, the Montreal Cognitive Assessment test; SCD-Q, Subjective Cognitive Decline Questionnaire;*

a *post hoc analyses showed a significant group difference between aMCI and HC;*

b *post hoc analyses showed a significant group difference between aMCI and SCD;*

c *post hoc analyses showed a significant group difference between SCD and HC;*

* *p < 0.05;*

** *p < 0.01;*

*** *p < 0.001;*

*aMCI, amnestic mild cognitive impairment; SCD, subjective cognitive decline; HC, healthy control*.

### Altered DFC Variability in the Triple Networks in Patients With SCD and aMCI

**Figure 2:**
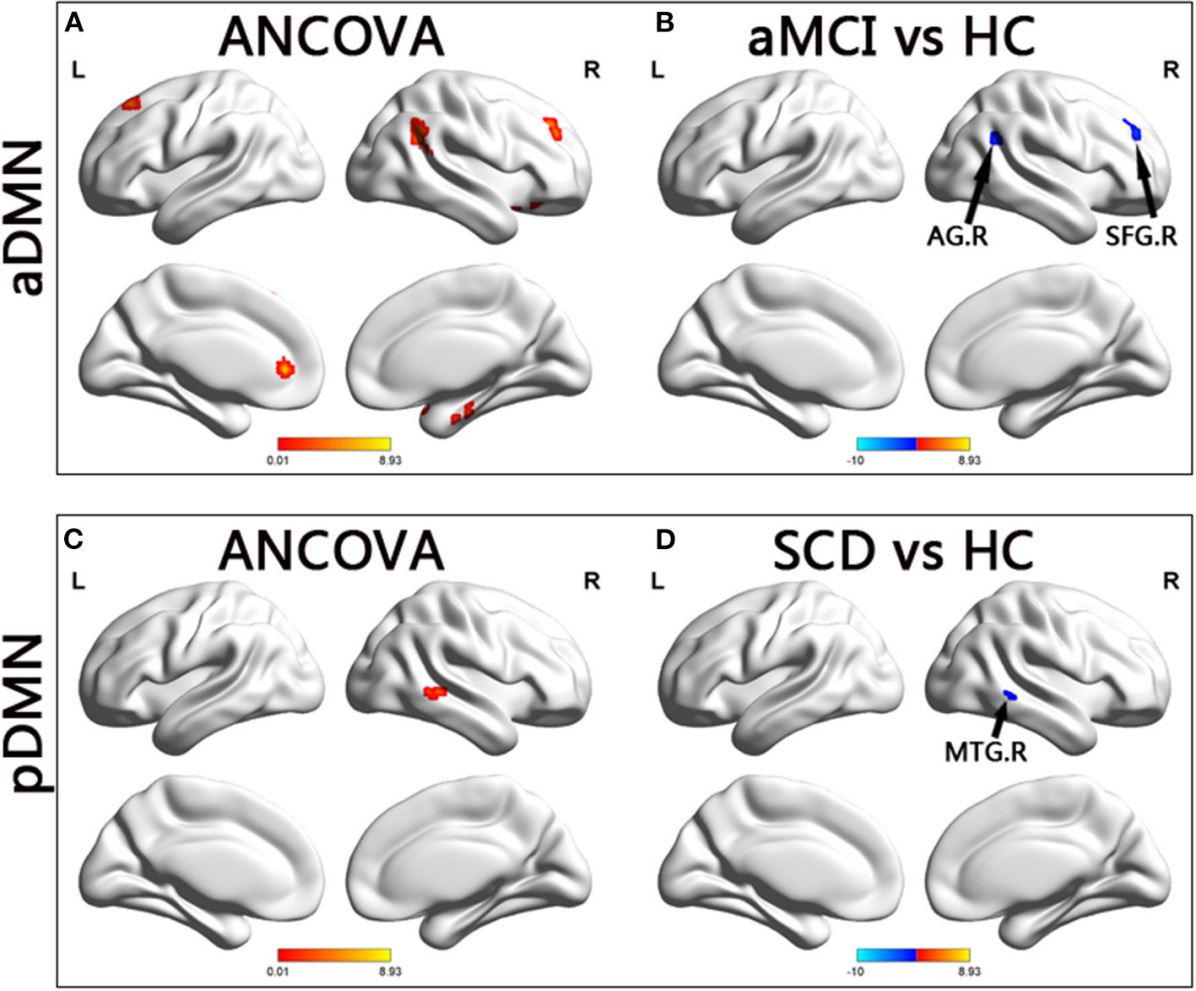
Brain regions exhibiting significant differences in dynamic functional connectivity variability within default mode network. **(A,C)** Brain regions showing significant differences in dynamic functional connectivity variability within the anterior default mode network and posterior default mode network across three groups, including SCD, aMCI, and HC (*p* < 0.05, the cluster size > 19 voxels). **(B,D)** Results of *post hoc* analysis in voxel-wise analysis (TFCE-FWE corrected, cluster size > 9, *p* < 0.05). aDMN, anterior default mode network; pDMN, posterior default mode network; aMCI, amnestic mild cognitive impairment; SCD, subjective cognitive decline; HC, healthy controls; AG, angular gyrus; SFG, superior frontal gyrus; MTG, middle temporal gyrus; R, right.

In the aDMN subnetwork, the ANCOVA results showed significantly altered DFC variability among the three groups, including the right parahippocampal gyrus, right inferior frontal gyrus (IFG), left anterior cingulum gyrus, left caudate, right angular gyrus, right superior temporal gyrus, and bilateral superior frontal gyrus (SFG). Compared with the HC group, the aMCI group showed decreased DFC variability in the right angular and right SFG (TFCE-FWE corrected, *p* < 0.05, cluster number > 9 voxels). All results were obtained with age, gender, and years of education as covariates (Table 2 and Figure 2).

**Table 2:** The difference of dynamic functional connectivity variability in default mode network across three groups.

**Region(aal)**	**Peak MNI coordinate**			**F/t**	**Cluster number**
	**x**	**y**	**z**		
**Anterior default mode network**					
**ANCOVA**					
R parahippocampal gyrus	21	−18	−30	6.3284	20
R inferior frontal gyrus	36	24	−24	7.0609	42
B anterior cingulum gyrus	−6	39	3	8.9345	22
L caudate	−9	6	−3	7.232	38
R angular gyrus/superior temporal gyrus	60	−51	21	6.6394	79
R superior frontal gyrus	21	39	27	7.4796	40
L superior frontal gyrus	−21	33	54	7.2538	21
**aMCI vs. HC**					
R angular gyrus	42	−51	24	−3.0653	18
R superior frontal gyrus	24	39	33	−3.6477	18
**Posterior default mode network**					
**ANCOVA**					
R middle temporal gyrus	66	−45	−3	6.9386	21
**SCD vs. HC**					
R middle temporal gyrus	66	−45	0	−3.1797	10
**Salience network**					
**ANCOVA**					
R Hippocampus	39	−27	6	3.8555	20
R superior temporal gyrus	51	−24	6	3.1027	34
L hippocampus	−18	−18	−15	6.3901	49
R inferior frontal gyrus	45	39	−6	7.1029	47
L insula/putamen	−45	12	3	11.4039	252
L superior temporal gyrus	−48	−39	18	6.4033	57
R inferior frontal gyrus	45	15	15	6.6237	28
**SCD vs. HC**					
L putamen	−30	−3	−3	3.6342	15
L insula	−45	12	3	4.1688	53
**aMCI vs. HC**					
L putamen	−27	12	−3	4.0464	11
**Executive control network**					
**ANCOVA**					
R middle frontal gyrus	33	54	9	7.1346	38
L inferior frontal gyrus	−39	9	24	6.3027	59
R middle frontal gyrus	45	36	21	5.0147	64
L middle frontal gyrus	−42	45	18	7.732	39
R inferior parietal lobule	54	−42	42	8.0083	32
**SCD vs. HC**					
L middle frontal gyrus	−48	42	21	3.8698	22
**aMCI vs. HC**					
R middle frontal gyrus	30	57	6	−3.3649	11
L inferior frontal gyrus	−36	15	15	3.7362	14

*The x, y, z coordinates are the primary peak locations in the MNI space. Cluster size > 19 voxels in ANCOVA analysis, p < 0.05; Cluster size > 10 voxels in post hoc test, p < 0.05, TFCE-FWE corrected; L, left; R, right; B, bilateral; aMCI, amnestic mild cognitive impairment; SCD, subjective cognitive decline; HC, healthy control*.

In the pDMN subnetwork, the ANCOVA showed significantly altered DFC variability in the right middle temporal gyrus (MTG) in the three groups. Compared with the HCs, the patients with SCD showed significant decreased DFC variability in the right MTG (TFCE-FWE corrected, *p* < 0.05, cluster number > 9 voxels). All results were obtained with age, gender, and years of education as covariates (Table 2 and Figure 2).

**Figure 3:**
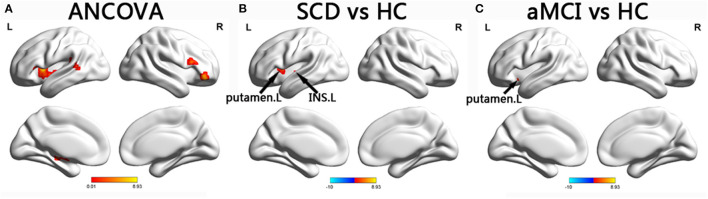
Brain regions exhibiting significant differences in dynamic functional connectivity variability within salience network. **(A)** Brain regions showing significant differences in dynamic functional connectivity variability within the salience network across three groups, including SCD, aMCI, and HC (*p* < 0.05, the cluster size > 19 voxels). **(B,C)** Results of *post hoc* analysis in voxel-wise analysis (TFCE-FWE corrected, cluster size > 9, *p* < 0.05). aDMN, anterior default mode network; pDMN, posterior default mode network; aMCI, amnestic mild cognitive impairment; SCD, subjective cognitive decline; HC, healthy controls; INS, insula; L, left.

In the SN, the ANCOVA showed significantly altered DFC variability in the left hippocampus, right IFG, left insula, left putamen, left STG, and right IFG. Compared with the group with HC, the group with aMCI showed increased DFC variability in the left putamen while the group with SCD showed increased DFC variability in the left putamen and left insula (TFCE-FWE corrected, *p* < 0.05, cluster number > 9 voxels). All results were obtained with age, gender, and years of education as covariates (Table 2 and Figure 3).

**Figure 4:**
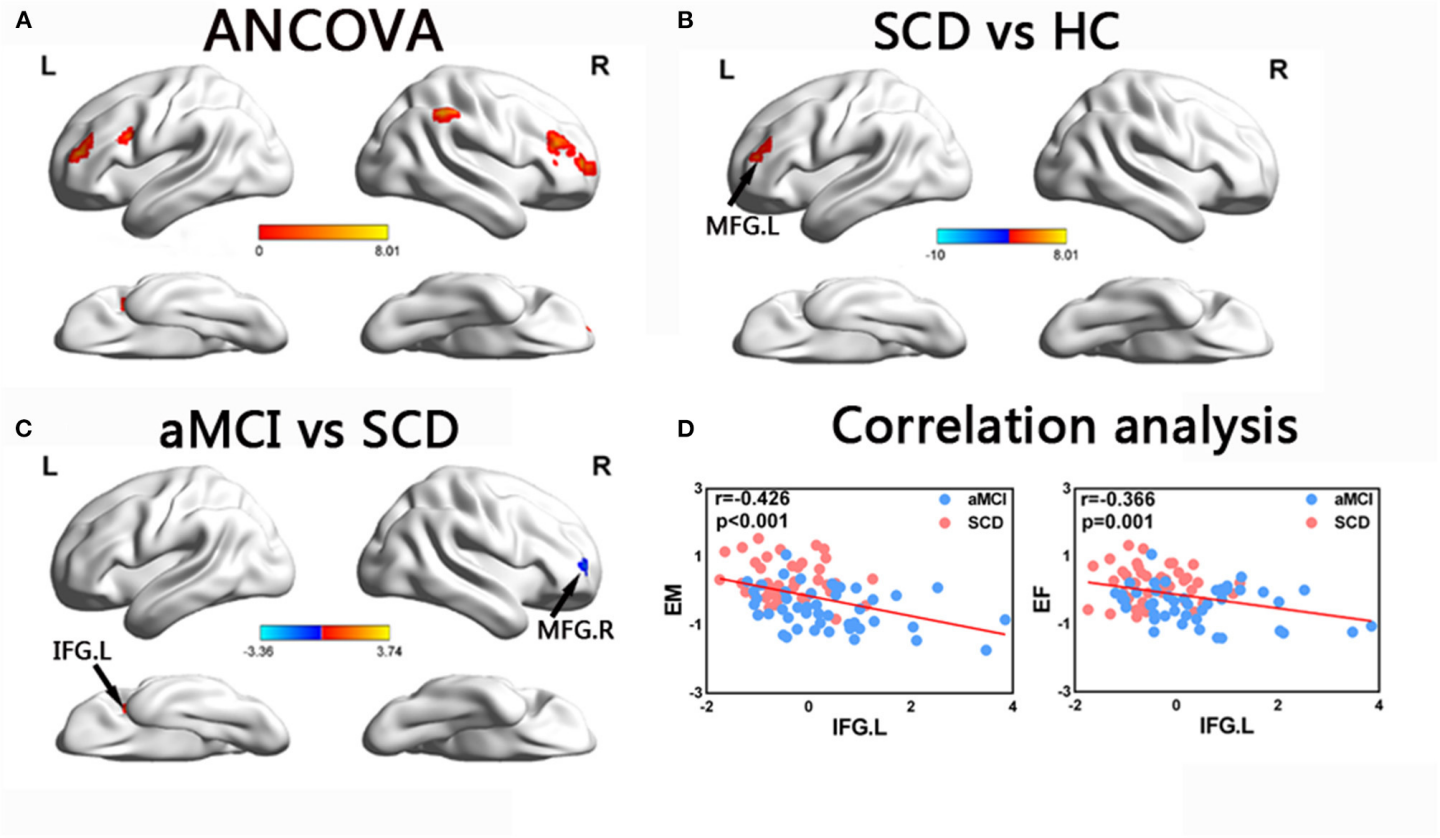
Brain regions exhibiting significant differences in dynamic functional connectivity variability within executive control network and the correlation with cognitive function. **(A)** Brain regions showing significant differences in dynamic functional connectivity variability within the executive control network across three groups, including SCD, aMCI, and HC (*p* < 0.05, the cluster size > 19 voxels). **(B,C)** Results of *post hoc* analysis in voxel-wise analysis (TFCE-FWE corrected, cluster size > 9, *p* < 0.05). **(D)** Results of associations between altered dynamic functional connectivity variability and cognitive function. Age, gender, and years of education were used as covariates of results (Bonferroni corrected, *p* < 0.05). aDMN, anterior default mode network; pDMN, posterior default mode network; aMCI, amnestic mild cognitive impairment; SCD, subjective cognitive decline; HC, healthy controls; MFG, middle frontal gyrus; IFG, inferior frontal gyrus; EM, episodic memory; EF, executive function; R, right; L, left.

In the ECN, the ANCOVA showed significantly altered DFC variability in the bilateral middle frontal gyrus (MFG), left IFG, and right inferior parietal lobule. Compared with the HCs, the group with SCD showed increased DFC variability in the MFG. Compared with SCD, aMCI showed decreased DFC variability in the right MFG, while increased DFC variability in left IFG (TFCE-FWE corrected, *p* < 0.05, cluster number > 9 voxels). All results were obtained with age, gender, and years of education as covariates (Table 2 and Figure 4).

### Behavioral Significance of Altered DFC Variability Within the Triple Networks in Patients With SCD and aMCI

The correlation analysis showed that in the groups with SCD and aMCI, altered DFC variability in the left IFG of the ECN was significantly negatively correlated with EM (*r* = −0.421, *p* < 0.001) and EF (*r* = −0.382, *p* < 0.001) (Bonferroni-corrected, *p* < 0.05). Age, gender, and years of education were used as covariates for all these results (Figure 4).

### Diagnosis and Classification of SCD and aMCI Using Logistic Regression Analysis

**Figure 5:**
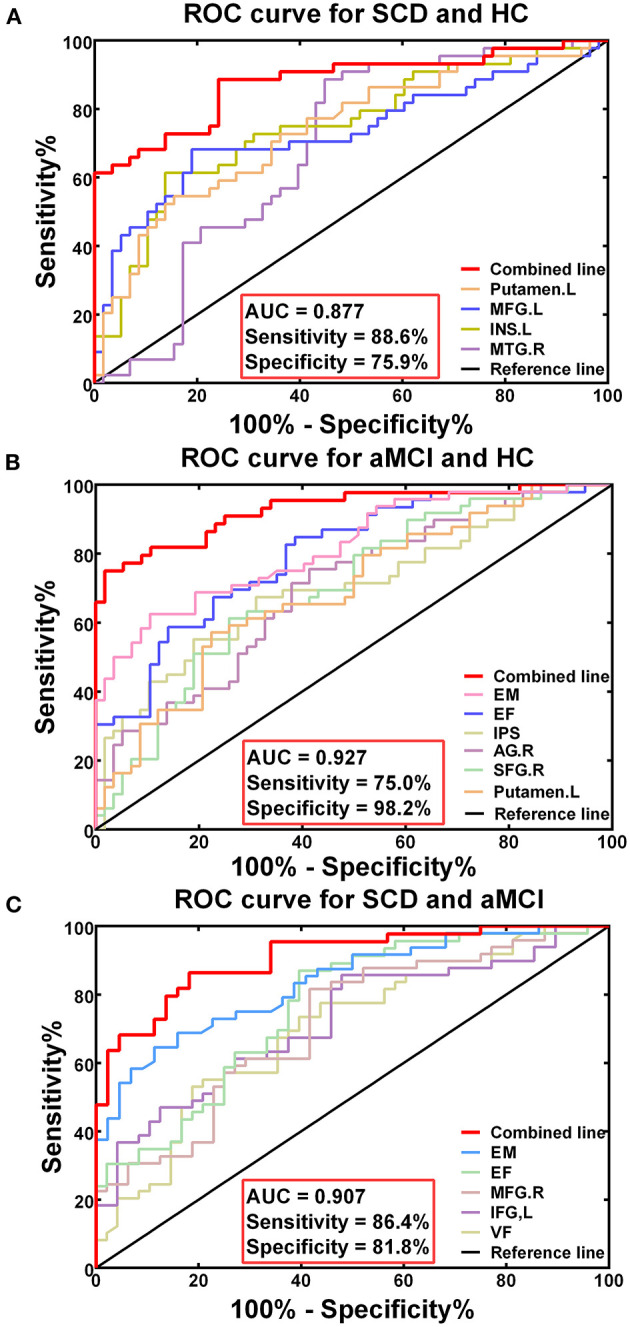
Diagnosis and differentiation of SCD and aMCI based on ROC analysis. **(A)** The ROC curve showing the classification of SCD and HC; **(B)** The ROC curve showing the classification of aMCI and HC; **(C)** The ROC curve showing the classification of aMCI and SCD.

The receiver-operating characteristic (ROC) curve of each altered index is shown in Figure 5. The best-fitting model was based on the multivariable models, combining altered DFC variability and decreased cognitive function. The AUC in the groups with SCD and HC based on the multivariable model was.877, with 88.6% sensitivity and 75.9% specificity (*p* < 0.001). In the groups with aMCI and HC, the AUC based on the multivariable model was.927, with 75.% sensitivity, and 98.2% specificity (*p* < 0.001). The AUC of the groups of SCD and aMCI based on the multivariable model was.907, with 86.4% sensitivity and 81.8% specificity (*p* < 0.001).

## Discussion

To the best of our knowledge, the present study was the first to analyze DFC variability in patients with SCD and aMCI based on the triple-network model and the association with cognitive decline. The primary findings of the study were that DFC variability within the triple networks in patients with SCD and aMCI had varying degrees of change. Moreover, altered DFC variability within the ECN was significantly correlated with cognitive performance in patients with SCD and aMCI. Most importantly, altered DFC variability, combined with the triple-network model, can serve as an important biomarker for their higher efficiency in the diagnosis of SCD and aMCI.

The present study showed that the DFC variability within the triple networks, including the DMN, SN, and ECN, was changed to different degrees in patients with SCD and aMCI. The DMN can be divided into the aDMN and pDMN, each of which has been considered to function independently in a wide range of cognitive tasks. Specifically, the aDMN is involved in self-referential mental idealization, while the pDMN is involved in EM retrieval (Xue et al., 2020). In the present study, patients with SCD patients showed decreased DFC variability in the right MTG within the pDMN compared with HCs, whereas patients with aMCI showed decreased DFC variability in the right angular gyrus and right SFG within the aDMN. The impaired brain regions are involved in language processing functions (angular gyrus), spatial orientation (angular gyrus), motor planning and executive (SFG) function, and visual information processing (MTG). This might mean that impairment in the DMN may lead to extensive cognitive decline. Moreover, a prior static FC study indicated that the FC of the aDMN first increased and then decreased with the progression of AD spectrum disease, which was consistent with our results, showing that DFC variability of the aDMN was decreased in patients with aMCI compared with HCs, while the patients with SCD remained stable (Xue et al., 2020). Notably, previous DFC studies demonstrated that higher DFC variability in brain regions may reflect greater complexity and greater information processing ability (Marusak et al., 2017). Decreased DFC variability may indicate the decreased information processing ability of patients with SCD and aMCI (Marusak et al., 2017). The decreased DFC variability within the DMN subnetworks of the patients with SCD in the present study means that patients with SCD already had a tendency toward impaired information processing. In addition, the patients with SCD showed altered DFC variability, mainly in the pDMN, whereas the patients with aMCI showed altered DFC variability mainly in the aDMN, which seemed to confirm the specificity of DFC variability within the DMN of patients with AD spectrum disorders.

In our study, the groups with SCD and aMCI both showed increased DFC variability in the left putamen within the SN, while the group with aMCI additionally showed increased DFC variability in the left insula within the SN. The putamen is part of the neostriatum, which was identified as one of the first brain areas affected by amyloid deposition in healthy elderly people (Rodriguez-Vieitez et al., 2016). Previous studies indicated that the putamen was involved in working memory and probabilistic learning and might be an appropriate clinical biomarker of neurodegenerative disease (Bellebaum et al., 2008; Looi et al., 2012). Research studies reported that a decline in the amplitude of low frequency fluctuations and volume of the putamen were significantly related to cognitive decline in patients with AD spectrum disorders (de Jong et al., 2008; Ren et al., 2016). The insula, a major region of the SN, is believed to play an important role in the maintenance of memory performance in the early stage of AD spectrum disorders (Lin et al., 2017). One study suggested that the left insula had the higher node degree and participation coefficient in the brain network and was associated with EM (Xue et al., 2020). The increased DFC variability of the SN in patients verified the “brain reserve” hypothesis that the enhanced FC of the SN in SCD and aMCI might be a compensatory mechanism for decreased DMN function, which resists amyloid protein deposition and maintains relatively normal cognitive function (Cohen et al., 2009; Menon and Uddin, 2010).

Our results showed altered DFC variability within the ECN in patients with SCD and aMCI. The ECN, with the prefrontal lobe as the core, plays an important role in the regulation of cognition and behavior, the integration of perception and memory information, and working memory (Petersen et al., 2019). The MFG and IFG are responsible for executive cognitive function and working memory. The present research found that SCD showed increased DFC variability in the left MFG compared with HCs, whereas patients with aMCI showed decreased DFC variability in the right MFG compared with the patients with SCD. This might indicate that DFC variability decreased as AD spectrum disorder progressed, representing a gradual decline in information-processing ability.

Taken together, we can speculate that patients with SCD and aMCI have a common and unique disruption in the triple networks. The triple networks are involved in a wide range of cognitive tasks through direct or indirect means. The disruption of any network of the triple networks will result in aberrant goal-related stimuli and internal psychological events (Sridharan et al., 2008). Previous research findings suggested that abnormal organization and function of the triple networks were prominent features of neuropsychiatric diseases. However, the specific changes in static FC within the triple networks of patients with SCD and aMCI were inconsistent. For example, some research claimed that aMCI showed increased static FC in the SN, while several reported disrupted static FC in the SN (Brier et al., 2012; Uddin, 2015; Chen et al., 2016b). One possible reason for the inconsistent results may be that the FC pattern was dynamic rather than static during the entire rsfMRI scan, leading to different FC patterns in different scan periods (Wang et al., 2020). Therefore, our study confirmed that the DFC of the triple networks was disrupted in patients with SCD and aMCI, suggesting that DFC analysis can be used to complement and verify static FC analysis.

The present study showed observably negative associations between altered DFC variability in the left IFG and cognitive domains in patients with SCD and aMCI, including EM and EF. The results demonstrated that disruption of DFC was significantly related to declining cognition performance in patients with SCD and aMCI. As the EM and EF were impaired, DFC variability in patients with SCD and aMCI increased in the left IFG. Moreover, the patients with aMCI exhibited higher DFC variability with the EM and EF impairment compared with patients with SCD. This might mean that the increased DFC variability in the left IFG was to compensate for the impairment of EM and EF in the progression of preclinical AD spectrum disorders. Furthermore, EF refers to the cognitive process of goal-oriented behavior from goal formulation to successful execution and the processing of results (Miller and Cohen, 2001; Diamond, 2013). The correlation between altered DFC variability in the left IFG within the ECN and EF confirmed why the ECN is widely used to investigate the mechanism of altered EF in patients (Brown et al., 2019). Interestingly, patients with SCD and aMCI showed significant correlations between EM and altered DFC variability within ECN. A previous study suggested that EM deficits in patients with aMCI were associated with the right dorsolateral prefrontal cortex functional network (Yuan et al., 2016). Our results provided new evidence for the interaction between impaired EF and memory impairment. Taken together, the study suggested that DFC in SCD and aMCI was disrupted, which extended the current understanding of the functional network and showed the importance of evaluating changes in DFC in patients with preclinical AD spectrum disorders.

The most significant finding in the current study was that the best-fitting model in diagnosing and characterizing SCD and aMCI was based on multivariable models. They combined altered DFC variability within the triple networks and declining cognitive function. It can be seen that the multivariable models had higher AUC values with high sensitivity and specificity compared with univariate models. The model was highly specific for aMCI with 98.2% specificity, so the risk of false-positive errors was very low, suggesting that DFC analysis could be a reliable potential biomarker for diagnosing patients with aMCI. Specifically, DFC variability in the left putamen played a vital role in the diagnosis of SCD, whereas DFC variability of the right angular gyrus played a major role in the diagnosis of aMCI due to its higher AUC values. Meanwhile, DFC variability in the right MFG and left IFG played dominant roles in the differentiation of SCD from aMCI. That distinction might provide additional information in research on specific brain region changes in SCD and aMCI. Additionally, research has indicated that the classification accuracy of static FC was lower than that of DFC because time-averaged analysis could not account for microscopic changes in brain states (Bassett et al., 2013; Allen et al., 2014; Cordova-Palomera et al., 2017). Studies have shown that DFC provided significantly more behavioral information than static FC (Cordova-Palomera et al., 2017; Liegeois et al., 2019). Thus, such reliable methods will have more value for the early detection of AD-related pathology.

## Limitations

Several limitations of the present study showed are acknowledged. First, the patient sample was small, which may have reduced the generalizability of the results. To avoid this problem, we applied a non-parametric permutation test to control the false-positive rate. Moreover, our research group is continuously recruiting new volunteers, and the NBH-ADsnp database is constantly updated, which means we will further verify our conclusions in the future. Secondly, we collected only 8-min-long data on each participant in the current study, resulting in inadequate results. We will take advantage of longer fMRI scan times, such as several hours, to improve the DFC variability estimates in a future study. Lastly, the lack of longitudinal research made it impossible to explore disease transformation in depth. Our research team is following up on the recruited volunteers regularly, and we plan to further explore the longitudinal changes in DFC in the future.

## Conclusion

The current study revealed common and specific DFC variability abnormalities within the triple networks of patients with SCD and aMCI. Moreover, altered DFC variability in the left IFG within the ECN was significantly correlated with cognitive decline, including EM and EF. More importantly, the best-fitting model in diagnosing and differentiating SCD and aMCI was a multivariable model that combined altered DFC variability (right MTG, left putamen, left insula, and left MFG in distinguishing patients with SCD and HCs; right AG, right SFG, and left putamen in distinguishing patients with aMCI and HCs; and right MFG and left IFG in distinguishing patients with SCD and aMCI) with declined cognitive function. Therefore, our findings suggest that the DFC variability analysis, combined with the triple-network model, can be used as a potential biomarker of preclinical AD spectrum disorders and may help us to understand abnormal cognitive function.

## Data Availability Statement

The data analyzed in this study is subject to the following licenses/restrictions: The datasets analyzed in this article are not publicly available. Requests to access the datasets should be directed to Jiu Chen, ericcst@aliyun.com.

## Ethics Statement

The studies involving human participants were reviewed and approved by the responsible Human Participants Ethics Committee of the Affiliated Brain Hospital of Nanjing Medical University. The patients/participants provided their written informed consent to participate in this study. Written informed consent was obtained from the individual(s) for the publication of any potentially identifiable images or data included in this article.

## Author Contributions

CXu, WQ, CXi, and JC designed the study. CXu, WQ, CXi, JC, QY, GH, HG, and JR collected the data. CXu analyzed the data and prepared the manuscript. All authors contributed to the article and approved the submitted version.

## Funding

This study was supported by the National Natural Science Foundation of China (No. 81701675); the Key Project supported by Medical Science and Technology Development Foundation, Nanjing Department of Health (No. JQX18005); the Cooperative Research Project of Southeast University-Nanjing Medical University (No. 2018DN0031); the Key Research and Development Plan (Social Development) Project of Jiangsu Province (No. BE2018608); and the Innovation and Entrepreneurship Training Program for College Students in Jiangsu Province (Nos. 201810312061X; 201910312035Z).

## Conflict of Interest

The authors declare that the research was conducted in the absence of any commercial or financial relationships that could be construed as a potential conflict of interest.

## Publisher's Note

All claims expressed in this article are solely those of the authors and do not necessarily represent those of their affiliated organizations, or those of the publisher, the editors and the reviewers. Any product that may be evaluated in this article, or claim that may be made by its manufacturer, is not guaranteed or endorsed by the publisher.
