# Supplementary material for: Disrupted Dynamic Functional Connectivity in Distinguishing Subjective Cognitive Decline and Amnestic Mild Cognitive Impairment Based on the Triple-Network Model
Source: Front Aging Neurosci. 2021 Sep 17;13:711009. doi: 10.3389/fnagi.2021.711009 (PMC8484524; doi:10.3389/fnagi.2021.711009)
Supplement: Supplementary file 1 [file Data_Sheet_1.doc]

**Disrupted dynamic functional connectivity in distinguishing subjective cognitive decline and amnestic mild cognitive impairment based on the triple-network model**

Chen Xue^1^, Wenzhang Qi^1^**,** Qianqian Yuan^1^, Guanjie Hu^2^, Honglin Ge^2^, Jiang Rao^3^, Chaoyong Xiao^1,2*^, Jiu Chen^2,4*^

^1^Department of Radiology, the Affiliated Brain Hospital of Nanjing Medical University, Nanjing, 210029, China

^2^Institute of Brain Functional Imaging, Nanjing Medical University, Nanjing, 210029, China

^3^Department of Rehabilitation, the Affiliated Brain Hospital of Nanjing Medical University, Nanjing, 210029, China

^4^Institute of Neuropsychiatry, the Affiliated Brain Hospital of Nanjing Medical University, Nanjing, Jiangsu, 210029, China

***Running Title:*** Salience network in SCD and aMCI.

***Correspondence to**:

**Chaoyong Xiao,** Department of Radiology, the Affiliated Brain Hospital of Nanjing Medical University, No.264, Guangzhou Road, Gulou District, Nanjing, Jiangsu, 210029, China. Email: [xchaoyong@163.com](mailto:xchaoyong@163.com).

**Jiu Chen,** Institute of Neuropsychiatry, Institute of Brain Functional Imaging, the

Affiliated Brain Hospital of Nanjing Medical University, No.264, Guangzhou Road,

Gulou District, Nanjing, Jiangsu, 210029, China. E-mail: [ericcst@aliyun.com](mailto:ericcst@aliyun.com)

# Supporting Information

### Supplementary Method

### S.1 NBH-ADsnp database

Data used in this study were obtained from the Nanjing Brain Hospital-Alzheimer’s Disease (AD) Spectrum Neuroimaging Project (NBH-ADsnp) database (in-home website: [http://192.168.8.100](http://192.168.8.100/)) (Nanjing, China). NBH-ADsnp was derived from an AD Spectrum Neuroimaging Project that was launched in January 2018 by the Institute of Brain Functional Imaging, the Affiliated Brain Hospital of Nanjing Medical University (Nanjing, China). Prof. Jiu Chen, PhD, MD, from the Affiliated Brain Hospital of Nanjing Medical University, served as the principal investigator of NBH-ADsnp. NBH-ADsnp was initiated by Dr. Jiu Chen and Dr. Xiangrong Zhang and was named by Dr. Jiu Chen's research group (discussed by Chen Xue, Guanjie Hu, Wenwen Xu, Wan Liu, Wenzhang Qi, Siyu Wang, Jiani Xu, Shanshan Chen, and finally verified by Jiu Chen and Xiangrong Zhang).NBH-ADsnp is an observational study which includes cross-sectional and longitudinal follow-up components. The goal of NBH-ADsnp is to identify early neuroimaging biomarkers of preclinical Alzheimer’s Disease (AD) spectrum {subjective cognitive decline (SCD), amnestic mild cognitive impairment (aMCI), non-amnestic mild cognitive impairment (naMCI), and AD}, to predict disease progression of individuals within the preclinical AD spectrum, and to provide imaging-based targets for individualized intervention to prevent disease deterioration from preclinical stages to the eventually progressed AD. Initially, several hundreds of elderly individuals in NBH-ADsnp, who were all Han Chinese and right-handed, were recruited from hospitals and local communities by advertising and by means of broadcasting. This database used a standardized clinical evaluation protocol that included a medical history interview, neurologic examination, a battery of neurocognitive assessments, and a resting-state MRI scan (T1, T2, 3D T1, DTI, and BOLD) for all participants (normal controls (CN), SCD, naMCI, aMCI, and AD). All subjects and their study partners completed the informed consent process, and study protocols were reviewed and approved by the responsible Human Participants Ethics Committee of the Affiliated Brain Hospital of Nanjing Medical University (No. 2018-KY010-01, No. 2020-KY010-02, No. ChiCTR1900022287, and No. ChiCTR2000034533).

The general eligibility, inclusion, and exclusion criteria for NBH-ADsnp subjects were as follows:

The inclusion criteria for the participants are: 1) right-handed Chinese Han patients aged between 50 and 80 years old; 2) Secondary school education or higher; 3) no history of taking psychoactive drug; 4) no history of serious brain diseases, such as brain tumor, brain infarction, brain hemorrhage, and severe brain injury; 5) no history of disease which could influence brain function, such as neuropathy, psychosis, thyroid dysfunction and systemic disease.

The inclusion criteria of HC were: 1) no memory complaints; 2) normal cognitive performance matched with age and education level; 3) Clinical Dementia Rating (CDR) = 0.

The inclusion criteria of SCD were based on the published SCD research criteria proposed by the Subjective Cognitive Decline Initiative (SCD-I): 1) always complained of memory problems; 2) Subjective Cognitive Decline Questionnaire (SCD-Q) > 5; 3) normal cognitive performance of age- and education-matched norms; 4) CDR = 0.

The inclusion criteria of aMCI were as follows: 1) patients complained of memory impairment of at least 3 months or relatives confirmed that the memory impairment last for more than 3 months; 2) impaired objective memory performance was based on the one of the following conditions: a. two neuropsychological tests in the episodic memory function that were within ≤ 1.0 Standard Deviation (SD) of age-adjusted norms; b. one neuropsychological test in the episodic memory function and the others tests in the other cognitive domain, including visual spatial function, executive function, and information processing speed were within ≤ 1.0 Standard Deviation (SD) of age-adjusted norms; 3) normal overall cognitive function evidenced by CDR = 0.5, Mini-mental State Examination (MMSE) score ≧ 24, Activities of Daily Living assessment-20 (ADL-20) ≦ 23, Mattis Dementia Rating Scale-2 (MDRS-2 ≧ 120), Hamilton Depression Rating Scale (HAMD) ≦ 7; 4) no dementia.

Detailed exclusion criteria for all subjects were described in our previously published studies: 1) a past history of stroke (modified Hachinski Ischemic Scale score > 4), alcoholism, head injury, brain tumors, Parkinson’s disease, epilepsy, encephalitis, major depression (excluded by HAMD), or other neurological or psychiatric illnesses (excluded by clinical assessment and case history); 2) major medical illness (e.g., cancer, anemia, thyroid dysfunction, syphilis, or HIV); 3) severe visual or hearing loss; 4) unable to complete neuropsychological tests or with a contraindication for MRI, and 5) T2-weighted MRI showing major changes in white matter (WM), infarction, or other lesions (two experienced radiologists analyzed the scans). None of the patients used any medications.

### S.2 Neuropsychological assessments for the NBH-ADsnp database

Neurocognitive assessments were as described in our previously published studies. The general cognitive functions data included the MMSE, the ADL, the MDRS-2, the MoCA, the SCD-Q, the CDR, and the HAMD. The episodic memory data contained the Auditory Verbal Memory Test-20min-delayed recall (AVLT-20-min DR), the Auditory Verbal Memory Test – recognition (AVLT-R), the Logical Memory Test -20 min delayed recall (LMT-20-min DR), and the Rey Complex Figure Test 20min delayed recall (CFT-20-min DR). The executive function data were derived from the Category Verbal Fluency Test (VFT) (including the VFT-animals and the VFT-objects), the Digit Span Test backward (DST-backward), part B of the Trail Making Test (TMT-B), part C of the Stroop Test (Stroop C), and Semantic Similarity. The information processing speed data were obtained from the Symbol Digit Modalities Test (DSST), part A of the Trail Making Test (TMT-A), part A and B of the Stroop Test. The visuospatial function data were extracted from the Rey Complex Figure Test (CFT) and the Clock Drawing Test (CDT). The individual raw score of each neuropsychological test was transformed to normalized Z scores. Subsequently, the normalized Z score was averaged to calculate the composite Z score of each cognitive domain.

### S.3 Image acquisition for the NBH-ADsnp database

The magnetic resonance imaging (MRI) data were collected using a 3.0 Tesla Verio Siemens scanner with an 8-channel head-coil in the Affiliated Brain Hospital of Nanjing Medical University. Resting-state functional images were acquired when participants were instructed to rest with their eyes open, to not fall asleep, and to not think of anything in particular. The gradient-echo echo-planar imaging (GRE-EPI) sequence included 240 volumes. The parameters were: repetition time (TR) = 2,000ms, echo time (TE) = 30ms, number of slices = 36, thickness = 4.0 mm, gap = 0 mm, matrix = 64× 64, flip angle (FA) = 90◦, field of view (FOV) = 220mm × 220mm, acquisition bandwidth = 100kHz, and voxel size = 3.4 × 3.4 × 4 mm^3^.

High-resolution T1-weighted images were acquired by 3D magnetization-prepared rapid gradient-echo (MPRAGE) sequence. The parameters were: TR = 1,900 ms, TE = 2.48 ms, inversion time (TI) = 900 ms, number of slices = 176, thickness = 1.0 mm, gap = 0.5 mm, matrix = 256 × 256, FA = 9◦, FOV = 256 mm × 256 mm, and voxel size = 1 × 1 × 1 mm^3^.
